# Memory-Augmented Spatio-Temporal Transformer for Robust Traffic Flow Forecasting

**DOI:** 10.3390/biomimetics11030170

**Published:** 2026-03-02

**Authors:** Puqing Hu, Chunjiang Wu, Chen Wang, Xin Yang, Zhibin Li, Tinghui Chen, Shijie Zhou

**Affiliations:** 1 School of Information and Software Engineering, University of Electronic Science and Technology of China, Chengdu 610054, China; 202112090809@std.uestc.edu.cn (P.H.); 202411090919@std.uestc.edu.cn (X.Y.); sjzhou@uestc.edu.cn (S.Z.); 2 School of Software Engineering, Chengdu University of Information Technology, Chengdu 610225, China; 3250707014@stu.cuit.edu.cn (C.W.); lizhibin@cuit.edu.cn (Z.L.); chenth@cuit.edu.cn (T.C.)

**Keywords:** traffic flow forecasting, spatio-temporal representation learning, graph attention networks, long-term temporal dependencies, neural prediction models

## Abstract

Accurate traffic flow prediction plays a critical role in intelligent transportation systems, supporting traffic management, congestion mitigation, and efficient utilization of road resources. Advances in neural network-based methods, particularly graph neural networks (GNNs) and attention-based models, have demonstrated strong capability in modeling spatio-temporal traffic dynamics. However, existing approaches still face notable challenges: GNN-based models often rely on static adjacency matrices, limiting their ability to capture dynamic and long-range spatial dependencies, while attention-based models usually involve complex architectures and heavy reliance on large-scale pre-training data. To address these limitations, this study proposes a novel traffic flow prediction model that integrates a learnable memory tensor into an attention-based framework. The introduced memory mechanism provides persistent global context for modeling long-term temporal dependencies in an end-to-end manner, enabling efficient and dynamic spatio-temporal representation learning with a lightweight architecture. Extensive experiments on multiple real-world traffic datasets demonstrate that the proposed model achieves superior prediction accuracy and robustness compared with existing baselines. The proposed approach offers a new perspective for memory-enhanced spatio-temporal modeling and provides valuable insights for traffic forecasting and related intelligent transportation applications.

## 1. Introduction

With the acceleration of urbanization and the advancement of intelligent transportation systems, accurately predicting traffic flow has become one of the key technologies for alleviating traffic congestion, optimizing travel routes, and enhancing road resource utilization [1,2,3,4]. High-quality traffic forecasting not only provides real-time decision support for urban traffic management but also holds significant application value in smart mobility, logistics distribution, energy scheduling, and other fields [5,6,7,8,9]. Consequently, researching predictive models capable of fully capturing the spatio-temporal dependency features of traffic has emerged as a hot topic in the field of transportation computing and intelligent systems [10].

In recent years, leveraging advancements in deep learning, neural network-based traffic forecasting methods have demonstrated significant advantages in modeling complex nonlinear relationships. Early works employed convolutional neural networks (CNNs) for traffic flow prediction, such as STResNet [11], which utilized 2D CNNs to model the spatial dependencies and residual structures of traffic networks, capturing long-term dependencies. Research predominantly employs graph neural networks (GNNs) to model traffic topology. For instance, STGCN [12] achieves joint modeling of spatial and temporal features by combining graph convolution with sequence modeling. Subsequently, ASTGCN [13] introduced an attention mechanism to enhance the model’s ability to represent spatio-temporal structures.

Although existing methods have achieved certain performance improvements, the aforementioned work and other related studies highlight the following issues:

(a) CNN-based approaches typically employ regular grids or fixed convolutional kernels, assuming fixed neighborhood relationships. This approach is ill-suited for the irregular topology of road networks and lacks sufficient modeling capability for long-range dependencies, making it difficult to adapt to complex dynamic traffic changes [11].
(b) GNN-based methods tend to rely heavily on static adjacency matrices, capturing only local spatial dependencies within fixed structures. They struggle to model interactions between distant or dynamically changing nodes [12].
(c) Many current models rely on large-scale data for training or pre-training. Under constrained conditions such as small cities or newly deployed sensor sections, data is often limited or sparse, significantly degrading the performance of data-hungry models [14].

From a biomimetics perspective, robust prediction in dynamic environments is a hallmark of biological intelligence: living systems continuously integrate noisy sensory inputs with accumulated experience and selectively deploy attention mechanisms to anticipate forthcoming states. Motivated by this principle, we view traffic flow forecasting as a bio-inspired information-processing task and design MFormer to leverage an adaptive attention mechanism enhanced by a learnable memory tensor, enabling persistent contextual recall during spatio-temporal prediction.

Specifically, this work introduces a learnable, node-wise Memory Tensor that serves as a compact external memory bank shared across all encoder layers. Each node is associated with a memory vector that is optimized end-to-end together with the forecasting network. Instead of relying on expensive pre-training or very deep temporal stacks, the Memory Tensor provides a persistent global context that can be injected into the attention computation, enabling the model to recall long-term patterns and stabilize predictions under sparse or noisy observations. This design is lightweight (the memory size is a tunable hyperparameter) and can be trained directly on target datasets without any additional self-supervised objectives. Unlike conventional methods that rely heavily on pre-training or static graph structures, the proposed model can accurately and efficiently predict dynamic traffic flow in complex urban environments. By integrating a learnable memory tensor within an attention-based framework, the model captures both short-term temporal fluctuations and long-term spatial correlations, enabling it to adapt to time-varying traffic propagation patterns and heterogeneous road network interactions. The main contributions of this paper are summarized as follows:

• Adaptive Memory Tensor Integration: This study designs a novel memory tensor module that adaptively encodes spatial and temporal dependencies in traffic data. This component enhances the attention mechanism by dynamically updating context representations according to evolving traffic conditions.
• Attention-based Architecture for Spatiotemporal Learning: This study constructs an attention-driven prediction framework that efficiently fuses spatial correlations among road segments with temporal dynamics, enabling the model to focus on critical patterns relevant to future traffic states.
• Pre-training-free Prediction Framework: The proposed model eliminates the need for pre-training, achieving competitive or superior prediction performance through end-to-end training directly on traffic datasets.
• Comprehensive Experimental Validation: Extensive experiments on multiple real-world traffic datasets demonstrate that the proposed model outperforms several existing baselines in terms of prediction accuracy and generalization ability, particularly under time-varying traffic scenarios.

The remainder of this paper is organized as follows. Section 2 reviews related work on traffic flow forecasting and Transformer-based spatio-temporal modeling. Section 3 formally defines the traffic forecasting problem and introduces the notation used throughout the paper. Section 4 presents the proposed MFormer model in detail, including the data embedding strategy, spatio-temporal encoder design, and the memory-augmented attention mechanism. Section 5 reports extensive experimental results on multiple real-world datasets and provides comparative evaluations and ablation studies to demonstrate the effectiveness of the proposed approach. Finally, Section 6 concludes the paper and discusses potential directions for future research.

## 2. Related Work

### 2.1. Traffic Flow Forecasting

Early traffic flow forecasting primarily relied on statistical modeling and classical machine-learning methods. Time-series models such as the Autoregressive Integrated Moving Average (ARIMA) and vector autoregression (VAR) were used to capture trends and periodicity and to jointly model multiple sensors [15,16]. Meanwhile, the Kalman Filter was shown to be effective for real-time forecasting under noisy measurements [17]. State-space neural network formulations were also explored to combine dynamical system modeling with nonlinear function approximation for prediction in traffic systems [18]. Traditional machine learning baselines such as Support Vector Regression (SVR) and Random Forest (RF) can be competitive, particularly for short-horizon prediction or when training data are limited [16]. However, these approaches usually struggle to capture nonlinear spatio-temporal interactions and non-Euclidean spatial correlations in large-scale road networks.

In recent years, deep learning has gained widespread adoption in traffic flow forecasting due to its powerful nonlinear modeling capability. Convolutional neural networks (CNNs) were first introduced to process rasterized traffic representations and capture local spatial patterns, and graph neural networks (GNNs) later became a mainstream choice by explicitly modeling road-network topology and non-Euclidean spatial relations. More recently, attention mechanisms and transformer-style architectures have shown strong performance because they can flexibly capture dynamic, long-range spatio-temporal dependencies. To better model complex spatio-temporal interactions, many works emphasize learned (rather than fixed) graph structures and heterogeneity, including adaptive graph generation [19], dynamic graph convolution/recurrent frameworks [20,21], and heterogeneity/non-stationarity-aware modeling [22,23]. Recent surveys and benchmarks further summarize these trends and highlight open challenges such as generalization, robustness, and scalability [24,25]. In parallel, transformer-based traffic predictors continue to evolve from early multi-attention designs toward hierarchical and delay-aware attention [26,27] and mixed attention/convolution designs for efficiency [28]. Pre-training and multi-source fusion are also being explored to improve transferability across cities and sensor layouts [29,30]. Beyond traffic-specific models, progress on long-horizon sequence modeling suggests that learnable memory and interpretable attention can be beneficial for forecasting [31,32].

Building upon these works, this study proposes an adaptive, learnable memory tensor that captures global spatio-temporal dependencies to enhance learning in the model’s self-attention layer.

### 2.2. Transformer

The Transformer model was initially proposed to address sequence modeling and machine translation problems in Natural Language Processing (NLP) [33]. By abandoning traditional recurrent neural networks, it resolved issues of vanishing gradients and computational inefficiency in modeling long sequences, becoming the first model to fully leverage attention mechanisms for sequence data. Its core advantages include modeling global dependencies, strong parallel computing capabilities, and stable parameter updates, significantly enhancing performance in tasks like text translation and semantic modeling. The Transformer’s structure is highly modular, primarily composed of multi-head self-attention mechanisms stacked with feedforward neural networks, enabling efficient capture of complex dynamic relationships within sequences. This structural versatility has rapidly extended the Transformer’s application from NLP to image recognition, speech recognition, and even spatio-temporal sequence tasks like traffic flow prediction in recent years. Research indicates that while traditional CNN and GNN models excel at modeling spatial dependencies, they are often constrained by local window limitations in temporal modeling. In contrast, Transformers can flexibly model dependencies across arbitrary time scales [34]. The emerging wave of Transformer-based traffic flow prediction models demonstrates leading performance and stability on public datasets, establishing Transformers as an indispensable modeling paradigm in modern traffic forecasting.

## 3. Problem Definition

### 3.1. Definition of the Road Network

The road network is represented as a graph G=(V,E,A), where $V={\{v}_{1},v_{2},\cdots ,v_{N}\}$ denotes the set of *N* nodes, E⊆V×V is the set of edges, and A∈$R^{N\times N}$ is the adjacency matrix encoding the structural connectivity between nodes in the network.

### 3.2. Definition of the Traffic Flow Tensor

Let $X_{t}\in R^{N\times C}$ denote the traffic flow at time t of *N* nodes in the road network, where *C* denotes the number of traffic-related features per node, for example, *C* = 2 when the data includes inflow and outflow. The complete historical record over T time steps is denoted by a traffic tensor $X=(X_{1},X_{2},\cdots ,X_{T})\in R^{T\times N\times C}$.

### 3.3. Problem Formalization

The objective of traffic flow prediction is to estimate future traffic conditions based on past observations. Given the historical traffic tensor over the previous T time steps and the underlying network topology G, the task is to learn a predictive mapping f that forecasts traffic states for the next $T^{\prime }$ timesteps: $f([X_{t-T+1},\cdots ,X_{t};G])\to [X_{t+1},\cdots ,X_{{t+T}^{\prime }}]$.

## 4. Methods

**Figure 1:**
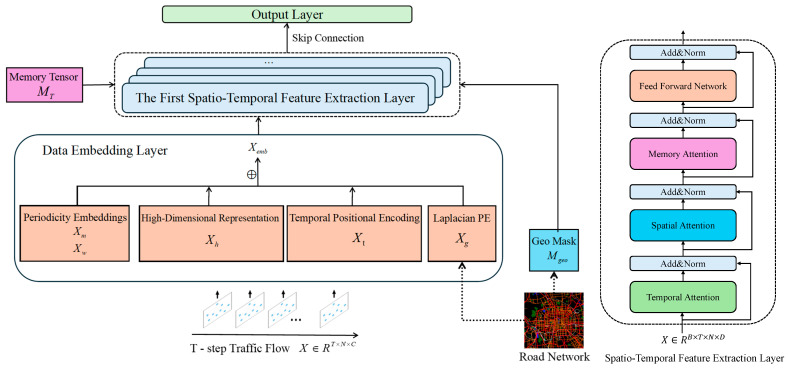
Overview of MFormer.

Figure 1 shows the framework of MFormer, which consists of a data embedding layer, stacked L spatial-temporal encoder layers, and an output layer. The following subsections describe each module in detail.

### 4.1. Data Embedding Layer

The data embedding layer converts the input into a high-dimensional representation. First, the raw traffic flow $X\in R^{T\times N\times C}$ is transformed into $X_{h}\in R^{T\times N\times d}$ through one fully connected layer, where C is the input dimension and d is the embedding dimension.

To enhance the model’s ability to perceive periodic features, we introduce two embeddings to cover the daily periodicity $\tau_{m}$ and weekly periodicity $\tau_{w}$. In this way, two periodicity embeddings are obtained, $X_{m}\in R^{T\times d}$ and $X_{w}\in R^{T\times d}$.

Graph Laplacian eigenvectors [35] are utilized for global road network modeling. First, the normalized Laplacian matrix is computed as:

(1) $$\Delta =I-D^{-1/2}AD^{-1/2}$$

where A is the adjacency matrix, D is the degree matrix, and I is the identity matrix. Second, the eigenvalue matrix Λ and the eigenvector matrix U are obtained by $\Delta =U^{T}\Lambda U$. Third, a fully connected layer is applied to the k smallest nontrivial eigenvectors to generate the global road network embedding $X_{g}\in R^{N\times d}$.

Following the original Transformer [33], a temporal positional encoding $X_{t}\in R^{T\times d}$ is employed to introduce position information of the input sequence.

Finally, the output of the data embedding layer is obtained by summing the above embedding vectors as:

(2) $$X_{emb}=X_{h}+X_{m}+X_{w}+X_{g}+X_{t}$$

### 4.2. Spatial-Temporal Encoder Layer

The spatio-temporal encoder layer converts the input $X_{emb}$ into complex and dynamic spatio-temporal features. The core of the encoder layer includes three components:

#### 4.2.1. Temporal Attention (TA)

A temporal attention mechanism is designed to capture dynamic global temporal dependencies in traffic data. Formally, for node n, the query, key, and value matrices are computed as:

(3) $$Q_{n}^{\left(T\right)}=X^{i}W_{Q}^{T},K_{n}^{\left(T\right)}=X^{i}W_{K}^{T},V_{n}^{\left(T\right)}=X^{i}W_{V}^{T}$$

Here $X^{i}$ is the input to the i-th layer of the encoder, and $W_{Q}^{T},W_{K}^{T},W_{V}^{T}\in R^{d\times d}$ are learnable parameters. Then, attention is applied along the temporal dimension to obtain the temporal dependencies between all time steps for node n as:

(4) $$A_{n}^{\left(T\right)}=\frac{(Q_{n}^{\left(T\right)}){\left(K_{n}^{\left(T\right)}\right)}^{T}}{\sqrt{d}}$$

Due to the inclusion of learnable query and key parameters, the global temporal dependencies $A_{n}^{\left(T\right)}\in R^{T\times T}$ is dynamic. Thus, the TA model can be adapted to capture the dynamic global temporal dependencies. Finally, the TA output is computed by multiplying the attention scores with the value matrix as:

(5) $$TA(Q_{n}^{\left(T\right)},K_{n}^{\left(T\right)},V_{n}^{\left(T\right)})=soft\max(A_{n}^{\left(T\right)})V_{n}^{\left(T\right)}$$

In this way, through the temporal attention in Equations (3)–(5), each time step interacts with all the time steps, and the dynamic global temporal dependencies $O_{TA}\in R^{T\times N\times d}$ can be obtained.

#### 4.2.2. Spatial Attention (SA)

We design a spatial mask attention mechanism to capture dynamic geographic dependencies in traffic data. Formally, for time step t, the query, key, and value matrices are computed as:

(6) $$Q_{t}^{\left(S\right)}=X^{i}W_{Q}^{S},K_{t}^{\left(S\right)}=X^{i}W_{K}^{S},V_{t}^{\left(S\right)}=X^{i}W_{V}^{S}$$

where $X^{i}$ is the input to the i-th layer of the encoder, and $W_{Q}^{S},W_{K}^{S},W_{V}^{S}\in R^{d\times d}$ are learnable parameters. Then, attention is applied along the spatial dimension to obtain the spatial dependencies among all nodes at time step t as:

(7) $$A_{t}^{\left(S\right)}=\frac{(Q_{t}^{\left(S\right)}){\left(K_{t}^{\left(S\right)}\right)}^{T}}{\sqrt{d}}$$

Due to the inclusion of the learnable parameters of query and key, the global spatial dependencies $A_{t}^{\left(S\right)}\in R^{N\times N}$ is dynamic. Thus, the SA model can be adapted to capture the dynamic global spatial dependencies. Finally, the output of the SA module can be obtained by the attention scores with the value matrix as:

(8) $$SA(Q_{t}^{\left(S\right)},K_{t}^{\left(S\right)},V_{t}^{\left(S\right)})=soft\max(A_{t}^{\left(S\right)})V_{t}^{\left(S\right)}$$

For the simple spatial attention in Equation (8), each node interacts with all nodes, which means that each node will interact with nodes that are not connected in the road network. As a result, the model is unable to accurately model the global information of the road network. Thus, a graph masking matrix $M_{geo}$ is required to help the model accurately model the global information of the road network.

(9) $$M_{geo}[i][j]=\left\{\begin{array}{c} \begin{array}{ccc} 0 & , & \lambda_{ij}\leq k \end{array}\\ \begin{array}{ccc} -\infty & , & \lambda_{ij}>k \end{array} \end{array}\right.$$

where $\lambda_{ij}$ is the hop count between node i and node j, which is calculated by the adjacency matrix, and k is a manually set threshold of hop count. Based on the value of $M_{geo}$ in Equation (9), a spatial masking mechanism is introduced as follows:

(10) $$MSA(Q_{t}^{\left(S\right)},K_{t}^{\left(S\right)},V_{t}^{\left(S\right)})=soft\max(A_{t}^{\left(S\right)}+M_{geo})V_{t}^{\left(S\right)}$$

In this way, through the spatial attention in Equation (10), each node interacts with adjacent nodes and itself, and the dynamic geographic spatial dependencies $O_{MSA}\in R^{T\times N\times d}$ can be obtained.

#### 4.2.3. Memory Attention(MA)

A global temporal attention mechanism is designed to enhance the model’s ability to perceive long-term temporal features. Formally, for node n, the query, key, and value matrices are computed as:

(11) $$Q_{n}^{\left(M\right)}=X^{i}W_{Q}^{M},K_{n}^{\left(M\right)}=X^{i}W_{K}^{M},V_{n}^{\left(M\right)}=X^{i}W_{V}^{M}$$

Then, to extract long-term temporal features, an adaptive memory tensor $M_{T}\in R^{T\times d}$ is introduced. Next, fully connected layers are used to transform the memory tensor into two new tensors $M_{T}^{K},M_{T}^{V}\in R^{T\times d}$, respectively. These two tensors are then used to update the key and value matrices in Equation (12):

(12) $$\begin{array}{l} {\bar{K}}_{n}^{\left(M\right)}=K_{n}^{\left(M\right)}+M_{T}^{K}\\ {\bar{V}}_{n}^{\left(M\right)}=V_{n}^{\left(M\right)}+M_{T}^{V} \end{array}$$

As the memory tensor continuously learns global temporal features, both the key and value matrices also acquire the ability to perceive global features. Based on Equations (4) and (5), dynamic long-term temporal features $O_{MA}\in R^{T\times N\times d}$ are obtained.

### 4.3. Output Layer

The output layer consists of three 1 × 1 convolutions:

(13) $$pre=W^{h_{2}}*(W^{t}*(\sum_{i=1}^{n}W_{i}^{h_{1}}*f_{i}+b_{i}^{h_{1}})+b^{t})+b^{h_{2}}$$

where $f_{i}$ is the output of the i-th layer of the encoder, ‘∗’ is the convolution operation, $W^{h_{2}}\in R^{d_{m}\times 1},W^{t}\in R^{T\times T_{p}},W_{i}^{h_{1}}\in R^{d\times d_{m}}$ are the convolution kernel, and $b^{h_{2}},b^{t},b_{i}^{h_{1}}$ are the bias corresponding to the convolution kernel.

The first 1 × 1 convolution maps the features to a higher dimension $d_{m}$ to enhance the model’s ability to represent traffic flow features. The second 1 × 1 convolution maps the features from the input length T to the output length $T_{p}$. And the third 1 × 1 convolution maps the features from dimension $d_{m}$ to output dimension 1.

Algorithm 1 summarizes the overall workflow of MFormer.
**Algorithm 1:** MFormer for Traffic Flow ForecastingInput: Historical traffic tensor X, time indices I, adjacency matrix A (or grid coordinates), input length $T_{\mathrm{IN}}$, output length $T_{\mathrm{OUT}}$, encoder depth L.Operation/* Initialization */1.Initialize learnable parameters (embeddings, attention projections, nonlinear feature transformation, and output head) and the Memory Tensor M.2.Compute Laplacian positional encoding and periodic embeddings; obtain embedded input $H^{0}$ using Equation (2).3.Construct the geographic masking matrix *G* using Equation (9) geo mask./* Spatial-Temporal Encoder (× *L*) */4.Set skip <-- 0.5.for l = 1 to L do6. Compute temporal attention using Equations (3)–(5) to obtain $H_{t}$.7. Compute spatial masked attention using Equations (6)–(10) with mask *G* to obtain $H_{s}$.8. Compute memory attention using Equations (11) and (12) and Equations (4) and (5) to obtain $H_{m}$ (long-term temporal features).9.   Apply nonlinear feature transformation + residual connection + normalization to obtain $H^{l}$.10. Update skip <-- skip + Conv1 × 1($H^{l}$).11.end for/* Output Head */12.Generate prediction $\hat{Y}$ using the output layer in Equation (13).13.Return $\hat{Y}$/* Operation Ending */Output: Predicted traffic tensor $\hat{Y}$ over the next $T_{\mathrm{out}}$ time steps.

## 5. Experiments

### 5.1. Datasets

The proposed MFormer is evaluated on six widely used real-world traffic datasets, covering both sensor-based road networks and grid-based urban mobility scenarios. The PeMS04, PeMS07, and PeMS08 datasets are collected from the California Performance Measurement System (PeMS) and consist of traffic flow measurements recorded by loop detectors deployed on highway networks. These datasets represent graph-structured traffic networks with irregular topologies and strong spatial dependencies. In contrast, NYCTaxi, CHIBike, and T-Drive are grid-based urban mobility datasets constructed by partitioning urban areas into regular spatial grids, where each grid cell aggregates inflow and outflow volumes within fixed time intervals. Together, these datasets cover diverse traffic patterns, spatial structures, and temporal resolutions, providing a comprehensive benchmark to evaluate the accuracy, robustness, and generalization ability of traffic flow forecasting models. All datasets used in this study are longitudinal time-series datasets collected through repeated measurements over extended periods, rather than single time-point observations. Traffic states are continuously recorded at fixed sampling intervals, which reflect the dynamic and evolving nature of urban traffic systems.

Table 1 summarizes the key statistics of all datasets employed in our experiments, including the number of nodes, edges, timesteps, sampling interval, and overall time range.

### 5.2. Baselines

To evaluate the performance of MFormer under different spatial structures, representative baselines are selected, and experiments are conducted separately on graph-based and grid-based datasets according to the modeling assumptions of each method.

This experimental design ensures a fair and meaningful comparison by avoiding the inappropriate application of models to incompatible spatial structures. Specifically, the selected baselines are categorized into four major groups:

- Grid-based models. This group includes STResNet [11], DMVSTNet [36], and DSAN [37], which are specifically designed for grid-structured urban mobility data. These models mainly employ convolutional or attention-enhanced convolutional operations over regular spatial grids, making them particularly suitable for datasets such as NYCTaxi and CHIBike, where traffic states are aggregated over fixed spatial cells. However, due to their reliance on predefined and regular neighborhood structures, these approaches are not explicitly tailored to irregular graph-structured road networks. As a result, they are primarily used as reference baselines for grid-based datasets rather than direct competitors on graph-based traffic networks.
- Time series prediction models. Classical time series methods, including VAR and SVR, are included as traditional statistical and machine learning baselines. These models focus primarily on temporal correlations and do not explicitly model spatial dependencies. Although their expressive power is limited compared to deep spatio-temporal models, they provide a useful lower-bound reference and help quantify the performance gains achieved by advanced neural architectures.
- Graph neural network-based models. This category consists of STGCN [12], MTGNN [14], DCRNN [38], STSGCN [39], STFGNN [40], STGODE [41], STGNCDE [42] and GWNET [43]. These methods explicitly model road network topology using graph convolution, diffusion processes, or neural differential equations. They represent the mainstream direction in traffic forecasting research and serve as strong baselines for evaluating MFormer’s ability to capture spatial dependencies in graph-structured data.
- Self-attention-based models. In addition, representative attention-driven models are included, namely GMAN [44], ASTGNN [22], TFormer [26], and STTN [45],. These methods leverage self-attention mechanisms to capture long-range spatio-temporal dependencies and are particularly relevant for comparison, as MFormer also adopts an attention-based architecture. Evaluating against these models highlights the effectiveness of the proposed memory-enhanced attention mechanism.

### 5.3. Experiment Settings

Data Processing. Following common practice in traffic flow forecasting, all datasets are split into training, validation, and test sets to ensure fair evaluation and reproducibility. For grid-based datasets, the data are divided at a ratio of 7:1:2, and the inflow and outflow of the past six time steps are used to predict the traffic conditions in the subsequent time window. This setting is consistent with prior studies on urban mobility prediction and reflects short-term forecasting scenarios. For graph-based datasets, a 6:2:2 split ratio is adopted, which is widely used in graph-based traffic prediction benchmarks. The model takes traffic observations from the past twelve time steps as input and predicts traffic flow for the next twelve time steps, enabling the evaluation of medium-horizon forecasting performance under realistic traffic dynamics.

Model Settings. All experiments are conducted on a machine equipped with an NVIDIA GeForce 4090 GPU(NVIDIA Corporation, Santa Clara, CA, USA). The hidden layer size d of MFormer is searched over {16, 32, 64, 128}.

The depth of the spatio-temporal feature extraction block is searched over {2, 4, 6, 8}. The feature dimension of the Memory Tensor is searched within {16, 32}. During model training, the AdamW optimizer [46] is employed with a learning rate of 0.001. The batch size is varied across {2, 4, 8, 16}, and training is conducted for 500 epochs. An early stopping mechanism is triggered when the best validation loss fails to decrease for 50 consecutive epochs. The optimal model is determined based on validation set performance.

We select the final architectural and training configuration based on validation performance, and the best hyperparameters for each dataset are summarized in Table 2. Regarding convergence, with early stopping and a maximum of 500 epochs, the selected best checkpoints are consistently reached within 97–255 epochs across the six datasets, indicating stable convergence behavior under our training protocol.

**Table 2:** Best hyperparameters of MFormer for each dataset.

Dataset	Average Best Epoch	Heads	Mem Size	Embedding	Enc Depth	Hidden	LapE Dim	Drop Path	Attn Drop	Batch
PeMS04	99	8	32	16	8	2	16	0.05	0.2	4
PeMS07	154	8	16	32	4	4	8	0.2	0.2	3
PeMS08	255	8	32	16	4	2	16	0.1	0.2	4
NYCTaxi	241	8	32	32	6	4	8	0.3	0.1	8
CHIBike	97	4	32	64	4	4	8	0.3	0.1	16
T-Drive	241	8	16	32	4	4	8	0.2	0.1	2

Evaluation Metrics. To comprehensively assess prediction accuracy, three widely used evaluation metrics are adopted: Mean Absolute Error (MAE), Mean Absolute Percentage Error (MAPE), and Root Mean Square Error (RMSE). These metrics jointly reflect absolute deviation, relative error, and sensitivity to large prediction errors. For grid-based datasets, traffic values below 10 are excluded during evaluation, following DMVSTNet [30]. Given the lower traffic volume in the CHIBike dataset, the threshold is adjusted to 5, consistent with PDFormer [27], ensuring fair and meaningful metric computation. We repeat all experiments ten times and report the average results.

### 5.4. Performance Comparison

**Figure 2:**
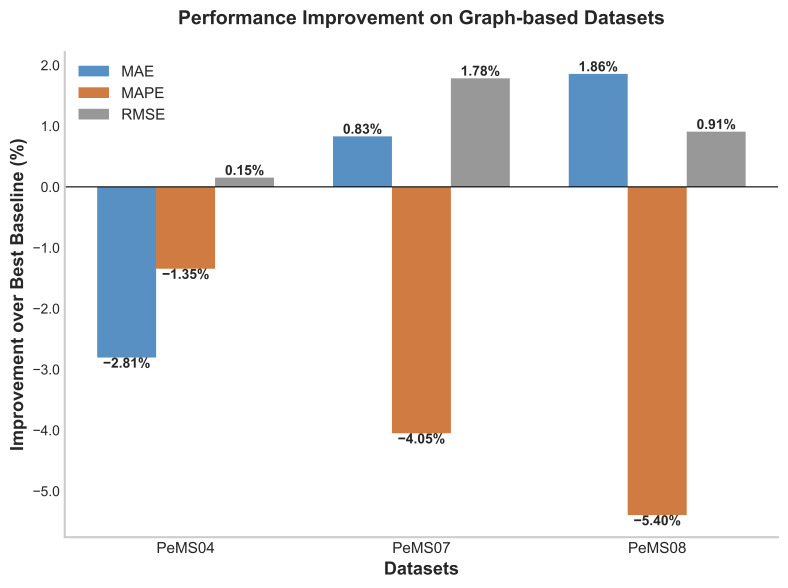
Performance improvement comparison on graph datasets; negative values indicate MFormer is not the best.

**Figure 3:**
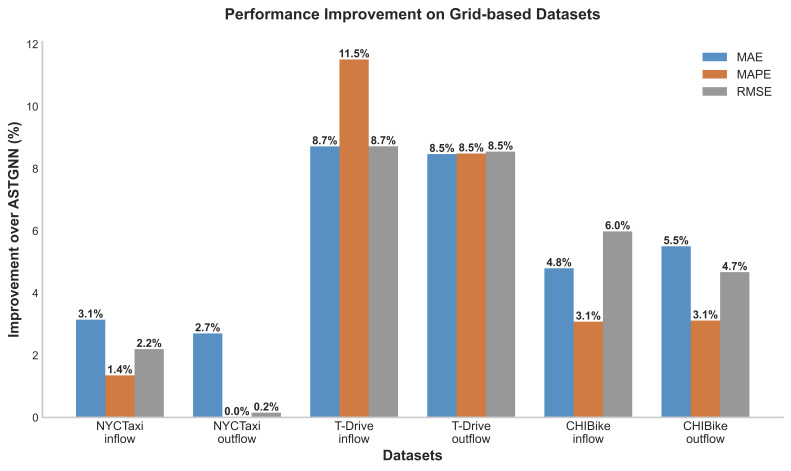
Performance improvement comparison on grid datasets.

Comparison results with baselines on graph-based and grid-based datasets are shown in Table 3, Table 4 and Table 5 and Table 6. Bolded results indicate the best performance, while underlined results represent the second-best performance. Based on the data in these tables, the experimental results indicate that:

(a) MFormer achieves the lowest MAE/MAPE/RMSE across all three grid-based datasets. Compared to the second-best model ASTGNN, MFormer achieves average improvements of 5.55%, 4.59%, and 5.04% in MAE, MAPE, and RMSE, respectively. Notably, MFormer significantly reduces prediction errors on the T-Drive and CHIBike datasets. Figure 2 and Figure 3 further illustrate the detailed improvement margins of MFormer relative to the best baseline. Within grid-based datasets, performance improvement varies by dataset characteristics: T-Drive shows the most significant gains, with MAE and RMSE exceeding 8%, and MAPE reaching 11.51% on inflow. This indicates that MFormer’s Memory Tensor mechanism offers superior modeling capabilities for trajectory data with high spatio-temporal sparsity, effectively learning long-term dependencies under sparse observations. Improvements on the NYCTaxi dataset range between 2% and 4%, while those on the CHIBike dataset range between 4% and 6%,demonstrating the model’s stability and universality across different urban transportation scenarios.
(b) Across three graph-based datasets, MFormer consistently demonstrates superior predictive performance. While slightly trailing ASTGNN in MAE and MAPE metrics, it achieves comprehensive leadership in RMSE—a metric more reflective of prediction stability—with improvement rates of 0.15%, 1.78%, and 0.91% on the PeMS04, PeMS07, and PeMS08 datasets, respectively. This result indicates that MFormer’s predictions exhibit smaller squared error deviations, stronger suppression of large errors and outliers, and more stable and reliable results, demonstrating its outstanding robustness.

Compared with time-series baselines, MFormer explicitly models spatial dependencies via spatial attention, which explains the consistent gains on network-wide forecasting tasks. Compared with GNN-based predictors, whose information propagation is effectively constrained by fixed-hop message passing, MFormer uses global attention to directly capture long-range spatial-temporal interactions without requiring deep diffusion steps, alleviating long-range dependency loss. Compared with attention-based models without external memory, the proposed Memory Tensor injects persistent node-wise global context into the attention computation, which stabilizes long-horizon prediction and suppresses large deviations; this is consistent with MFormer’s stronger RMSE performance on graph datasets and its larger improvements on sparse mobility data such as T-Drive.

**Table 6:** Performance on Graph-based Datasets.

Models	PeMS04			PeMS07			PeMS08		
	MAE	MAPE(%)	RMSE	MAE	MAPE(%)	RMSE	MAE	MAPE(%)	RMSE
VAR	23.750	18.090	36.660	101.200	39.690	155.140	22.320	14.470	33.830
SVR	28.660	19.150	44.590	32.970	15.430	50.150	23.250	14.710	36.150
DCRNN	22.737	14.751	36.575	23.634	12.281	36.514	18.185	11.235	28.176
STGCN	21.758	13.874	34.769	22.898	11.983	35.440	17.838	11.211	27.122
GWNET	19.358	13.301	31.719	21.221	9.075	34.117	15.063	9.514	24.855
MTGNN	19.076	12.961	31.564	20.824	9.032	34.087	15.396	10.170	24.934
STSGCN	21.185	13.882	33.649	24.264	10.204	39.034	17.133	10.961	26.785
STFGNN	19.830	13.021	31.870	22.072	9.212	35.805	16.636	10.547	26.206
STGODE	20.849	13.781	32.825	22.976	10.142	36.190	16.819	10.623	26.240
STGNCDE	19.211	12.772	31.088	20.620	8.864	34.036	15.455	9.921	24.813
STTN	19.478	13.631	31.910	21.344	9.932	34.588	15.482	10.341	24.965
GMAN	19.139	13.192	31.601	20.967	9.052	34.097	15.307	10.134	24.915
TFormer	18.916	12.711	31.349	20.754	8.972	34.062	15.192	9.925	24.883
ASTGNN	**18.601**	**12.630**	31.028	20.616	**8.861**	34.017	14.974	**9.489**	24.710
MFormer	19.123	12.800	**30.981**	**20.445**	9.220	**33.410**	**14.696**	10.001	**24.486**

Due to the strong forecasting performance of attention-based models, we further compare the computational cost of MFormer with representative attention-based baselines on the PeMS04 and NYCTaxi datasets. Table 7 reports the average training and inference time per epoch measured under the same hardware and implementation environment in this work. GMAN [44] and ASTGNN [22] adopt an encoder–decoder architecture, whereas STTN [45] replaces the encoder–decoder pipeline with a forward procedure. TFormer [26] performs only spatial attention, resulting in reduced attention computation along the temporal dimension. In contrast, MFormer employs a forward prediction pipeline with a stacked spatio-temporal attention encoder augmented by an adaptive Memory Tensor, and Table 7 shows that it achieves competitive training and inference efficiency among attention-based baselines while maintaining strong forecasting accuracy.

### 5.5. Ablation Study

To validate the effectiveness of the Memory Tensor in MFormer, we created two variants using MFormer:

(1) *w*/*o* MemTensor: This variant removes the Memory Tensor, meaning the model’s attention module loses the spatio-temporal adaptive enhancement provided by the Memory Tensor. (2) *w*/*o* GeoMask: This variant removes the geographic masking matrix, meaning each node attends to all nodes rather than only those closer to it. MFormer is comprehensively compared with the two variants.

**Figure 4:**
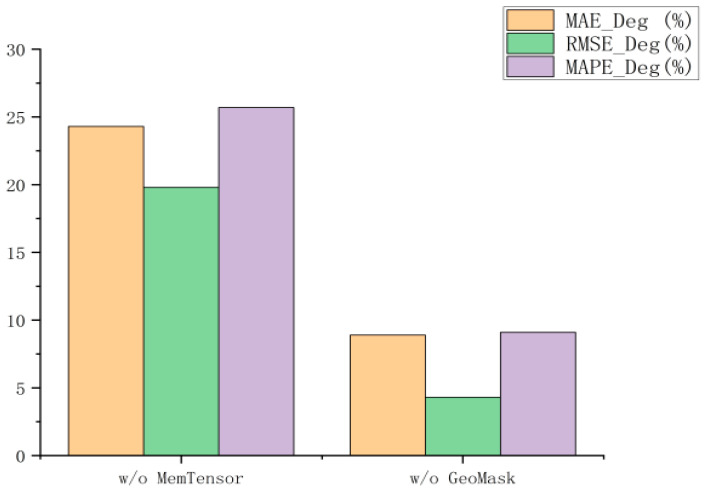
Performance degradation under ablation settings.

**Figure 5:**
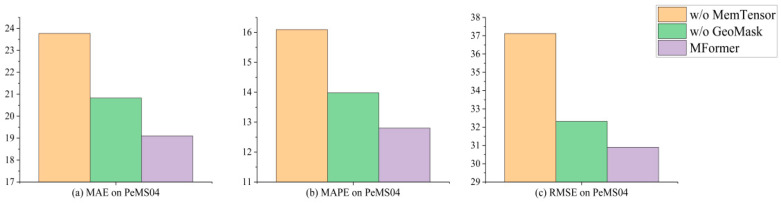
Ablation study on PeMS04.

Figure 4 quantifies the performance degradation when removing each component. Figure 5 shows the comparison results between MFormer and the two variants on the PeMS04 dataset. The results indicate that:

(a) MFormer achieves significant improvements over w/o MemTensor across all three metrics: RMSE is reduced by 19.8%, MAE is reduced by 24.3%, and MAPE is reduced by 25.7%. This is because the proposed Memory Tensor possesses strong adaptive learning capabilities and effectively learns complex spatio-temporal relationships in real traffic scenarios, thereby enhancing traffic flow prediction accuracy.

(b) The prediction performance of w/o GeoMask deteriorates compared to MFormer: RMSE increases by 4.3%, MAE by 8.9%, and MAPE by 9.1%. Although the degradation is relatively minor, it still indicates that the geographic masking has a positive effect on the model’s prediction performance.

(c) Comparing the performance of variants without MemTensor, without GeoMask, and MFormer reveals that removing Memory Tensor causes an average performance degradation of 23.3%, while removing GeoMask results in an average degradation of 7.5%. This significant disparity clearly demonstrates that the Memory Tensor is the core component of MFormer, with its adaptive learning capability playing a decisive role in model prediction accuracy. In contrast, GeoMask serves as a supplementary prior constraint, complementing the optimization of model performance.

## 6. Conclusions

This study proposes a novel traffic flow prediction model, MFormer. This model adaptively captures complex spatiotemporal features in real traffic and continuously learns during training. This not only significantly enhances the model’s ability to model long-term time series dependencies but also enables it to more flexibly handle dynamic spatial relationships and nonlinear variations within road networks. By organically integrating temporal attention, spatial attention, and memory attention mechanisms, MFormer constructs an end-to-end unified prediction model. This architecture simultaneously captures local short-term fluctuation patterns and global long-term evolutionary trends, thereby providing a more comprehensive feature representation for traffic state prediction. Experimental results on six real-world traffic datasets demonstrate that MFormer significantly outperforms mainstream baseline models across multiple evaluation metrics, exhibiting higher prediction accuracy, stronger generalization capabilities, and improved robustness. From a practical perspective, improved traffic forecasting accuracy may translate into tangible economic benefits, including reduced congestion duration, lower fuel consumption, improved fleet dispatch efficiency, and enhanced infrastructure utilization. Even modest error reductions can contribute to significant cost savings in large-scale urban transportation systems. Although a full economic simulation is beyond the scope of this work, the proposed approach provides a promising foundation for real-world intelligent transportation deployment.

Despite MFormer’s strong predictive performance, the following limitations remain:

(a) The static design of the Memory Tensor struggles to dynamically adapt to varying traffic patterns and sudden events across different time periods, since it is globally shared and optimized during training without time-conditioned updates;

(b) Sequential computation of triple attention mechanisms creates efficiency bottlenecks in large-scale networks, due to the quadratic complexity of attention operations along temporal and spatial dimensions.

(c) Insufficient model interpretability hinders understanding of the spatio-temporal patterns learned by each component and their decision-making mechanisms. This limitation arises because the triple-attention mechanism and Memory Tensor learn highly distributed and implicitly encoded representations without explicit semantic or physically grounded constraints, making their internal decision logic difficult to disentangle.

Future work focuses on three directions. First, future work explores dynamic and context-aware memory mechanisms. While MFormer currently employs a static learnable Memory Tensor to encode long-term patterns, future designs incorporate time-conditioned updates and event-aware routing to better adapt to abrupt traffic changes while retaining stable long-term regularities. More efficient large-scale inference strategies are investigated, such as sparse or factorized attention, cluster-based spatial routing, and caching of structural encodings, to reduce computational overhead and support real-time deployment on city-scale networks. Model interpretability is enhanced by developing memory attention visualization and attribution techniques, enabling clearer analysis of how memory representations capture recurring traffic patterns and improving the transparency and reliability of model predictions.

## Figures and Tables

**Table 1:** Statistics of the datasets.

Datasets	Nodes	Edges	Timesteps	Time Interval	Time Range
PeMS04	307	340	16,992	5 min	1 January 2018–28 February 2018
PeMS07	883	886	28,224	5 min	5 January 2017–31 August 2017
PeMS08	170	295	17,856	5 min	1 July 2016–31 August 2016
NYCTaxi	75 (15 × 5)	484	17,520	30 min	1 January 2014–31 December 2014
CHIBike	270 (15 × 18)	1966	4416	30 min	1 July 2020–30 September 2020
T-Drive	1024 (32 × 32)	7812	3600	60 min	1 February 2015–30 June 2015

**Table 3:** Performance on NYCTaxi Dataset.

Dataset	NYCTaxi					
Metrics	Inflow			Outflow		
Models	MAE	MAPE (%)	RMSE	MAE	MAPE (%)	RMSE
STResNet	14.492	14.543	24.05	12.798	14.368	20.633
DMVSTNet	14.377	14.314	23.734	12.566	14.318	20.409
DSAN	14.287	14.208	23.585	12.462	14.272	20.294
DCRNN	14.421	14.353	23.876	12.828	14.344	20.067
STGCN	14.377	14.217	23.86	12.547	14.095	19.962
GWNET	14.31	14.198	23.799	12.282	13.685	19.616
MTGNN	14.194	13.984	23.663	12.272	13.652	19.563
STSGCN	15.604	15.203	26.191	13.233	14.698	21.653
STFGNN	15.336	14.869	26.112	13.178	14.584	21.627
STGODE	14.621	14.793	25.444	12.834	14.398	20.205
STGNCDE	14.281	14.171	23.742	12.276	13.681	19.608
STTN	14.359	14.206	23.841	12.373	13.762	19.827
GMAN	14.267	14.114	23.728	12.273	13.672	19.594
TFormer	13.995	13.912	23.487	12.211	13.611	19.522
ASTGNN	13.844	13.692	23.177	12.112	13.602	19.201
MFormer	**13.409**	**13.507**	**22.668**	**11.785**	**13.601**	**19.172**

**Table 4:** Performance on T-Drive Dataset.

Dataset	T-Drive					
Metrics	Inflow			Outflow		
Models	MAE	MAPE (%)	RMSE	MAE	MAPE (%)	RMSE
STResNet	19.636	17.831	34.89	19.616	18.502	34.597
DMVSTNet	19.599	17.683	34.478	19.531	17.621	34.303
DSAN	19.384	17.465	34.314	19.29	17.379	34.267
DCRNN	22.121	17.75	38.654	21.755	17.382	38.168
STGCN	21.373	17.539	38.052	20.913	16.984	37.619
GWNET	19.556	17.187	36.159	19.55	15.933	36.198
MTGNN	18.982	17.056	35.386	18.929	15.762	35.992
STSGCN	23.825	18.547	41.188	24.287	19.041	42.255
STFGNN	22.144	18.094	40.071	22.876	18.987	41.037
STGODE	21.515	17.579	38.215	22.703	18.509	40.282
STGNCDE	19.347	17.134	36.093	19.23	15.873	36.143
STTN	20.583	17.327	37.22	20.443	15.992	37.067
GMAN	19.244	17.11	35.986	18.964	15.788	36.12
TFormer	18.823	16.91	34.47	18.883	15.674	35.219
ASTGNN	18.798	16.101	33.87	18.79	15.584	33.998
MFormer	**17.16**	**14.248**	**30.918**	**17.199**	**14.262**	**31.093**

**Table 5:** Performance on CHIBike.

Dataset	CHIBike					
Metrics	Inflow			Outflow		
Models	MAE	MAPE (%)	RMSE	MAE	MAPE (%)	RMSE
STResNet	4.767	31.382	6.703	4.627	30.571	6.559
DMVSTNet	4.687	32.113	6.635	4.594	31.313	6.455
DSAN	4.612	31.621	6.695	4.495	31.256	6.367
DCRNN	4.236	31.264	5.992	4.211	30.822	5.824
STGCN	4.212	31.224	5.954	4.148	30.782	5.779
GWNET	4.151	31.153	5.917	4.101	30.69	5.694
MTGNN	4.112	31.148	5.807	4.086	30.561	5.669
STSGCN	4.256	32.991	5.941	4.265	32.612	5.879
STFGNN	4.234	32.222	5.933	4.264	32.321	5.875
STGODE	4.169	31.165	5.921	4.125	30.726	5.698
STGNCDE	4.123	31.151	5.913	4.094	30.595	5.678
STTN	4.16	31.208	5.932	4.118	30.704	5.723
GMAN	4.115	31.15	5.91	4.09	30.662	5.675
TFormer	4.071	31.141	5.878	4.037	30.647	5.638
ASTGNN	4.068	31.131	5.818	3.981	30.617	5.609
MFormer	**3.873**	**30.173**	**5.47**	**3.762**	**29.664**	**5.347**

**Table 7:** Training and inference time per epoch comparison between attention-based models (Unit: s).

Dataset	PeMS04		NYCTaxi	
Model	Training	Inference	Training	Inference
GMAN	91.208	11.701	12.454	1.094
ASTGNN	34.798	5.872	10.508	0.900
STTN	28.652	13.247	5.250	0.511
TFormer	22.111	3.588	5.310	0.314
MFormer	50.626	8.574	6.650	0.396

## Data Availability

The datasets used in this study are available at https://gitee.com/origin115/memory-augmented-spatio-temporal-transformer-for-robust-traffic-flow-forecasting-datasets (accessed on 25 February 2026).
